# Case study on adverse reactions of dulaglutide: literature review

**DOI:** 10.3389/fphar.2026.1736083

**Published:** 2026-01-30

**Authors:** Yang Zhou, Weihao Jiang

**Affiliations:** 1 Department of Pharmacy, The Fifth People’s Hospital of Chengdu University of Traditional Chinese Medicine/Chengdu Fifth People’s Hospital, Chengdu, China; 2 Department of Pharmacy, Chengdu Institute of Brain Science Clinical Hospital, University of Electronic Science and Technology/Chengdu Fourth People’s Hospital, Chengdu, China

**Keywords:** adverse drug reactions, case, dulaglutide, literature, medication safety

## 1 Introduction

Glucagon like peptide-1 receptor agonists (GLP-1 RA), as a new generation of hypoglycemic drugs, can simulate the physiological effects of GLP-1 and have multiple clinical advantages such as lowering blood sugar, weight loss, and cardiovascular benefits (Chinese Medical Association Diabetes Branch, 2021).

Dulaglutide belongs to GLP-1RA and was approved for marketing in China in 2019 (Zheng et al., 2020). Common adverse drug reactions (ADRs) are mainly gastrointestinal reactions (Chinese Society of Endocrinology and and Chinese Society of Diabetes, 2020). At present, there are relatively few case reports on ADR. Based on existing case reports at home and abroad, this study aims to analyze the characteristics of ADR occurrence and development related to this drug, and provide reference for safe clinical use.

## 2 Data and methods

### 2.1 Source of information

Retrieve databases from China National Knowledge Infrastructure, Wanfang, and VIP PubMed、Web of Science. The Chinese search terms are “dulaglutide”, “adverse reactions”, and “case reports”; The English search terms are “dulaglutide”, “dulaglutide induced”, “dulaglutide related”, “adverse reaction”, and “case report”. The search period is from September 2014 to March 2025, excluding literature with unclear descriptions and duplicate cases. A total of 21 articles (Okiro et al., 2017; Taylor and Moody, 2018; Hamann et al., 2019; Rajput et al., 2018; Fukuda et al., 2019; Patel et al., 2019; Lee et al., 2021; Moore et al., 2024; Rzepka and Kaffenberger, 2020; Kalas et al., 2021; Butler et al., 2021; Kohli et al., 2021; Karakousis et al., 2021; Kyriakos et al., 2022; Samhani et al., 2024; Sonego et al., 2023; Vaccaro et al., 2023; Baker Khan et al., 2023; Shahbazi et al., 2023; Wang et al., 2023; Zhang et al., 2024) (involving 22 patients) were obtained that meet the criteria, including 20 English articles involving 21 patients; One Chinese literature article involving one patient.

### 2.2 Methods

The basic information of each ADR case study was counted, with information on gender, age, underlying disease, dulaglutide administration, and time of ADR occurrence, clinical presentation, management and regression of all patients.

## 3 Results

### 3.1 Basic information of patient

A total of 21 case studies containing 22 patients were included in this study. All of these cases were reported from 2017 to 2024, of which 10 were from the United States, 2 were from Ireland, 2 were from China, and individual cases from countries such as Denmark, France, and Greece were also included, and their basic profiles are shown in Table 1. The 22 patients included in the study consisted of 12 males and 10 females, and their underlying diseases were all diabetes mellitus, except for one case (Baker Khan et al., 2023), which was type 1 diabetes mellitus (T1DM) and one (Zhang et al., 2024) had latent autoimmune diabetes mellitus (LADA) in adults, all other patients had type 2 diabetes mellitus (T2DM). Common comorbidities in these patients included hypertension, hyperlipidemia, obesity, coronary artery disease, hyperuricemia, hyperthyroidism or disorders, chronic obstructive pulmonary disease (COPD), heart failure, coronary artery disease (CAD), and chronic obstructive pulmonary disease (COPD). The dosage of dulaglutide was 0.75 or 1.5 mg per week in all patients except for 6 (Hamann et al., 2019; Patel et al., 2019; Rzepka and Kaffenberger, 2020; Kohli et al., 2021; Vaccaro et al., 2023; Zhang et al., 2024)patients with incomplete information, 1 (Butler et al., 2021) patient with a dulaglutide dosing regimen of 2 mg per week and 1 (Samhani et al., 2024) patient with a dulaglutide dosing regimen of 3 mg per week. In addition, it is noteworthy that 4 (Rajput et al., 2018; Samhani et al., 2024; Baker Khan et al., 2023; Shahbazi et al., 2023) patients had ADRs that occurred only after the dosing dose had risen, and the original dosing dose did not contribute to the occurrence of the ADRs. The correlation between ADRs and the use of Dulaglutide in 22 patients was evaluated as “possible” according to the Measures for the Reporting and Monitoring of Adverse Drug Reactions. Most of the patients used 1-4 kinds of combination drugs, mainly hypoglycemic drugs, antihypertensive drugs, lipid regulating drugs and antiplatelet drugs. Common hypoglycemic agents are metformin, sulfonylurea hypoglycemic agents and insulin, common antihypertensive agents are amlodipine and bisoprolol, common lipid regulating agents are simvastatin and resuvastatin, and antiplatelet agents are mainly aspirin. Referring to the drug inserts of the above co-administered drugs, no significant interaction with Dulaglutide has been observed. However, maintenance or gradual reduction of insulin dose should be considered when using Dulaglutide, which may increase the risk of diabetic ketoacidosis if the dose is reduced or discontinued too rapidly. The gender and age distribution of the patients is shown in Table 2.

**TABLE 1:** Basic information of case study research.

Serial number	Year of publication	Lead author	Country to the research results belong
1 (Okiro et al., 2017)	2017	Julie Omolola Okiro	Ireland
2 (Taylor and Moody, 2018)	2018	Shawn R Taylor	United States
3 (Hamann et al., 2019)	2018	Carsten R Hamann	Denmark
4 (Rajput et al., 2018)	2018	Rajesh Rajput	India
5 (Fukuda et al., 2019)	2019	Gen Fukuda	Japan
6 (Patel et al., 2019)	2019	Anish Vinit Patel	United States
7 (Lee et al., 2021)	2020	Jayden Lee	United States
8 (Moore et al., 2024)	2021	Hannah E Moore	United States
9 (Rzepka and Kaffenberger, 2020)	2021	Polina V Rzepka	United States
10 (Kalas et al., 2021)	2021	M Ammar Kalas	United States
11 (Butler et al., 2021)	2021	Jared Butler	United States
12 (Kohli et al., 2021)	2021	Varun Kohli	United States
13 (Karakousis et al., 2021)	2021	Nikolaos Karakousis	Greece
14 (Kyriakos et al., 2022)	2022	Georgios Kyriakos	Spain
15 (Samhani et al., 2024)	2023	C Samhani	France
16 (Sonego et al., 2023)	2023	Benedetta Sonego	Italy
17 (Vaccaro et al., 2023)	2023	Christopher J Vaccaro	United States
18 (Baker Khan et al., 2023)	2023	Abu Baker Khan	Pakistan
19 (Shahbazi et al., 2023)	2023	Mohammad Shahbazi	United States
20 (Wang et al., 2023)	2023	Junwen Wang	China
21 (Zhang et al., 2024)	2024	Jiaming Zhang	China

**TABLE 2:** Distribution of the patients’gender and age [case (%), n = 22].

Age (Years old)	Male	Female	Total and percentage of the overall count
31–40	2	0	2 (9.09%)
41–50	1	2	3 (13.64%)
51–60	2	3	5 (22.73%)
61–70	2	3	5 (22.73%)
71–80	4	1	5 (22.73%)
80+	1	1	2 (9.09%)

### 3.2 Characteristics of ADR occurrence

The vast majority of patients experienced ADR within 15 months after medication (21 cases, 95.45%), with a higher incidence within 2 months after medication (19 cases, 86.36%). It is worth noting that in 3 patients (Rajput et al., 2018; Baker Khan et al., 2023; Shahbazi et al., 2023), the dosage of dulaglutide increased from 0.75 mg per week to 1.5 mg per week, and in 1 patient (Samhani et al., 2024), it increased from 1.5 mg per week to 3 mg per week. No adverse drug reactions occurred before the dosage doubled. The occurrence time and clinical manifestations of ADR are detailed in Table 3. The organs or systems most commonly affected by ADR are the skin and its appendages (6 cases, 27.27%), the digestive system (5 cases, 22.73%), and the circulatory system (5 cases, 22.73%). The organs/systems and clinical symptoms affected by ADR are shown in Table 4.

**TABLE 3:** Types and onset of adverse drug reactions (n = 22).

After the first dose (or after using a new treatment regimen)	Number of cases	Types of adverse drug reactions
Within 2 months	19	Myocardial infarction (1) Acute pancreatitis (2) Vaginal bleeding (1) Allergic reactions (including one rare allergic reaction, Kounis syndrome) (2) Ketoacidosis (3) Ischemic stroke (1) Rash (2) Acute kidney injury (1) Liver injury (1) Hypoglycemia (1) Vascular edema (1) Atrial fibrillation (1) Bullous pemphigoid (1) Pyoderma gangrenosum (1)
2–15 months	2	Bullous pemphigoid (1) Gastroparesis (1)
Over 15 months	1	Acute cholecystitis (1)

**TABLE 4:** ADR involved organs/systems and types of diseases (n = 22).

Affected organs/systems	Types of diseases	Number of cases (%)
Skin and its appendages	Rash (2) Bullous pemphigoid (2) Pyoderma gangrenosum (1) Allergic reactions (1)	6 (27.27%)
Digestive system	Acute pancreatitis (2) Gastroparesis (1) Liver injury (1) Acute cholecystitis (1)	5 (22.73%)
Circulatory system	Myocardial infarction (1) Kounis Syndrome (1) Ischemic stroke (1) Vascular edema (1) Atrial fibrillation (1)	5 (22.73%)
Reproductive system	Vaginal bleeding (1)	1 (4.55%)
Urinary system	Acute kidney injury (1)	1 (4.55%)
Endocrine system	Hypoglycemia (1)	1 (4.55%)
Others	Ketoacidosis (3)	3 (13.64%)

Among all 22 patients, 21 improved after discontinuation of medication or symptomatic supportive treatment, while 1 patient (Kohli et al., 2021) had a poor prognosis due to severe allergic reactions and developed ischemic brain injury. The ADR observed in this patient is Kounis syndrome (Han et al., 2024), also known as ST segment elevation acute coronary syndrome associated with allergies. It occurs due to allergic reactions to disease, food, medication, or environmental factors, which may be accompanied by rash, urticaria, skin itching, nausea, vomiting, wheezing, and angioedema; This disease has an impact on the coronary artery system, leading to the occurrence of acute coronary syndrome. At present, the academic community’s attention and research depth on this disease are clearly insufficient. Although there are some related reports, most of them are individual case reports. The control of ADR is symptomatic treatment, such as the use of antihistamines or glucocorticoids for allergic reactions, wound drainage for severe skin infections, the use of cardiovascular disease-related drugs for circulatory system symptoms, or surgical treatment for severe cases. It should be noted that two patients did not stop using dulaglutide. One case (Moore et al., 2024) had hypoglycemia due to not following the weekly dosage of 1.5 mg dulaglutide as prescribed by the doctor, but instead using 1.5 mg dulaglutide daily. After following the correct doctor’s advice and reducing insulin dosage appropriately, her symptoms improved significantly. The other patient (Butler et al., 2021) underwent cholecystectomy to treat acute cholecystitis induced by dulaglutide. The patient continued to use dulaglutide after surgery, and there was no recurrence of gallstones or other abdominal symptoms 8 months after the initial cholecystitis event. The performance, handling, and outcome of ADR are detailed in Table 5.

**TABLE 5:** Clinical manifestation, treatments and outcomes of ADRs.

Serial number	Clinical manifestations	Methods of treatment	Consequence
1 (Okiro et al., 2017)	Severe dehydration, low blood pressure, hypothermia, rapid heart rate	Discontinue dulaglutide immediately with IV saline and insulin, IV ceftriaxone	Relief to discharge after 6 days
2 (Okiro et al., 2017)	mildly impaired consciousness	Discontinue dulaglutide immediately and administer saline and insulin intravenously	Relief to discharge after 4 days
3 (Taylor and Moody, 2018)	kidney damage	Discontinue dulaglutide immediately	Significant improvement on day 10
4 (Hamann et al., 2019)	Rapidly expanding, painful, erythematous nodules on the trunk	Immediate discontinuation of dulaglutide with adalimumab and cyclosporine	Significant improvement after 2 months
5 (Rajput et al., 2018)	Headaches and blurred vision	Discontinue dulaglutide immediately and administer mannitol and low molecular heparin intravenously. Follow up with oral oral anticoagulants and antiplatelet agents	Significant improvement after 3 months
6 (Fukuda et al., 2019)	Pruritic erythematous lesions on the scalp and forearm skin, followed by extension of the pruritic lesions to both lower extremities	Immediate discontinuation of dulaglutide and injection of prednisolone and minocycline	The prognosis is good
7 (Patel et al., 2019)	Darkened urine, itching, epigastric pain, decreased appetite	Discontinue dulaglutide immediately	Significant improvement after 3 months
8 (Lee et al., 2021)	Severe nausea, intermittent vomiting, weakness, loss of appetite and palpitations	Immediate discontinuation of dulaglutide and treatment with diltiazem drip and electroconvulsive therapy	Relief to discharge after 1 day
9 (Moore et al., 2024)	hypoglycemia	Changing the frequency of dulaglutide injections to once a week and immediately discontinuing lysergic insulin and decreasing the dose of lysergic insulin	Patient’s condition improved significantly on day 18
10 (Rzepka and Kaffenberger, 2020)	Measles-like pinkish-red macules and papules all over the body	Discontinue dulaglutide immediately and use tretinoin cream application and prednisone	Rapid relief of symptoms
11 (Kalas et al., 2021)	Abdominal pain, nausea, vomiting	Discontinue dulaglutide immediately	Significant improvement after 4 weeks
12 (Butler et al., 2021)	Nausea, loss of appetite, progressive intensity, and pain in the right upper abdomen	Cholecystectomy (without discontinuation of dulaglutide)	Continued use of dulaglutide with no adverse effects
13 (Kohli et al., 2021)	Shortness of breath with a feeling of suffocation	Immediate discontinuation of dulaglutide and treatment of anaphylactic reactions with steroids, diphenhydramine, and epinephrine, with advanced cardiac life support after relapse of symptoms a few days after remission	End of treatment leaves sequelae of hypoxic brain damage
14 (Karakousis et al., 2021)	Mild swelling of the neck neck with moderate hives	Discontinue dulaglutide immediately	Rapid relief of symptoms
15 (Kyriakos et al., 2022)	Rash on arms and legs	Discontinue dulaglutide immediately and use betamethasone valerate cream coated with	Significant improvement after 2 weeks
16 (Samhani et al., 2024)	Itching, erythema, maculopapular rash on the abdomen at the injection site, and sleep disturbances	Immediate discontinuation of dulaglutide and treatment with antihistamines	Significant improvement after 6 days
17 (Sonego et al., 2023)	Mild erythematous rash progressing to maculopapular lesions	Immediate discontinuation of dulaglutide with prednisone and doxycycline	The prognosis is good
18 (Vaccaro et al., 2023)	Vaginal bleeding, severe fatigue, shortness of breath, decreased hemoglobin	Discontinue dulaglutide immediately and discontinue aspirin	The prognosis is good
19 (baker Khan et al., 2023)	Abdominal pain radiating to the back with nausea and vomiting, elevated lipase	Discontinue dulaglutide immediately and treat with painkillers	Relief to discharge after a few days
20 (Shahbazi et al., 2023)	Nausea, abdominal pain with mild epigastric tenderness	Immediate discontinuation of dulaglutide and rehydration with fluids, as well as bisacodyl and linaclotide	Relief to discharge after 3 days
21 (Wang et al., 2023)	Nausea, severe vomiting, chest pain	Immediate discontinuation of dulaglutide and continuous pumping of ondansetron injection, followed by coronary balloon dilatation and intra-aortic balloon counterpulsation	Relief to discharge after 7 days
22 (Zhang et al., 2024)	Severe nausea, vomiting, fatigue, and loss of appetite; recurrence of vomiting and dyspnea after initial hospital discharge	Immediate discontinuation of dulaglutide and vasodilator therapy	Significant improvement after 3 days

## 4 Discussions

### 4.1 Common ADR types using dulaglutide

The common ADRs in the drug instructions of Dulaglutide are gastrointestinal reactions, including nausea, vomiting, and diarrhea. In addition, there are conditions such as hypoglycemia, acute pancreatitis, increased heart rate, allergic reactions, aggravation of cholecystitis and atrial fibrillation. However, gangrenous pyoderma, measles like drug eruption, bullous pemphigoid, and the risk of liver damage associated with drugs that involve the skin are not included in their instructions and should be considered as new ADRs. Among them, gangrenous pyoderma and bullous pemphigoid have a more severe degree of occurrence and are considered as new severe ADRs.

### 4.2 ADR in relation to gender, age and dose and duration of medication administration

The gender difference of the cases included in this study was not significant, and the age was more than 50 years old (17 cases, 77.27%), which is consistent with the common age of onset of T2DM. With increasing age, the body’s hepatic and renal metabolic capacity decreases, which is one of the factors for the increase in ADR. The dosage of the drug in 14 patients in this study was 0.75–1.5 mg per week, which is a routine dosage. Although a study (Van et al., 2021) found no significant difference in the incidence of ADRs in patients after using dulaglutide at high doses (3.0 mg and 4.5 mg per week) compared to 1.5 mg per week, it is still recommended to use it according to the drug’s instructions and should not arbitrarily increase the dose. The results of this study showed that the time span of ADR occurrence of dulaglutide was relatively large, with an extreme value gap of more than 15 months. Therefore, it is important to closely monitor patients when starting Dulaglutide.

### 4.3 ADR accumulation system and clinical presentation

Skin and adnexa: It has been shown that dipeptidyl peptidase-4 (DPP-4) inhibitors are correlated with herpetic pemphigoid (Arai et al., 2018), and GLP-1RA may also damage the skin due to the expression of the GLP-1 receptor in cutaneous fibroblasts and keratin-forming cells (Gether et al., 2019). Therefore, GLP-1RA and DPP-4 inhibitors may have similar mechanisms for causing skin and adnexal damage, and it is recommended that patients should be specifically asked in detail about their past drug history and allergy history before administration. Circulatory system: GLP-1 receptors are found in the human pancreas, intestine and heart (Thompson and Trujillo, 2015). Animal experiments have shown that activation of GLP-1 receptors in the autonomic nervous system enhances the activity of the sympathetic nervous system and attenuates the activity of the parasympathetic nervous system. It has been suggested that overactivation of myocardial GLP-1 receptors may increase the risk of exacerbation of atrial fibrillation in patients with paroxysmal atrial fibrillation (Luo et al., 2020). In addition, regarding the formation of cerebral venous thrombosis may be related to dehydration due to nausea and vomiting in patients. Although the incidence of circulatory ADRs with dulaglutide is low, it still needs to be emphasized and should be used with caution in patients with previous arrhythmias. Digestive system: Gastrointestinal reactions are the most common type of ADR with Dulaglutide. According to a survey in China, gastrointestinal reactions were most pronounced in the first 2 weeks of treatment (Guo et al., 2020), and the dulaglutide drug insert also mentions a cumulative 104-week reported incidence of gastrointestinal adverse events when dulaglutide 0.75 mg and 1.5 mg were used weekly, respectively, including nausea (12.9% and 21.2%), diarrhea (10.7% and 13.7%), and vomiting (6.9% and 11.5%), mostly mild or moderate, so patients need to be evaluated for gastrointestinal disorders prior to administration. In addition, the drug delays gastric emptying and prolongs gallbladder contraction, which may increase the risk of gallbladder-related disorders.

### 4.4 Recommendations for pharmacovigilance of GLP-1RA analogs

An adverse reaction signal mining study based on the OpenFDA database (Dong and Wang, 2022) showed that GLP-1RA analogs were highly associated with ADRs such as pancreatitis, cholelithiasis, hypoglycemia, dizziness, urticaria, and injection site reactions. Liraglutide and exenatide had a higher incidence of digestive ADRs compared to dulaglutide. In addition, liraglutide had the highest incidence of acute kidney injury, exenatide had the highest incidence of hypoglycemia, and the use of dulaglutide was associated with a lower probability of ADRs involving the gastrointestinal, urinary, and endocrine systems compared with the former two. However, dulaglutide and exenatide were more prone to injection site reactions, such as localized augmentation and inflammation, compared to other types of GLP-1RAs. Taking into account the cases collected in this study and the actual clinical application, it is recommended that medical personnel start using dulaglutide at a small dose, pay attention to the patients’ previous drug history and allergy history before use, and promptly deal with ADRs when they are detected. Usually, patients with hepatic impairment do not need to adjust the dosage, but the monitoring of hepatic and renal functions should be strengthened during the use of the drug. In addition, the drug should be used with caution in patients with previous cardiac arrhythmias and acute and chronic renal impairment. If pancreatitis is suspected, it should be discontinued gradually. Dulaglutide should not be reused in patients with pancreatitis.

## 5 Conclusion

To sum up, dulaglutide, as a new hypoglycemic drug, provides a new choice for the treatment of diabetes, but its ADR cannot be ignored. The ADR caused by dulaglutide included in this study involves multiple systems, and some ADRs are not included in its drug instructions. With the widespread use of the drug, there may be more unknown ADRs. Medical staff need to continuously strengthen their understanding of dulaglutide ADR, especially in strengthening medication monitoring for patients with multiple comorbidities. Meanwhile, further research is needed on the mechanism of ADR of dulaglutide.

## Data Availability

The raw data supporting the conclusions of this article will be made available by the authors, without undue reservation.
